# Early development of a gonadal tumor in a patient with mixed gonadal dysgenesis

**DOI:** 10.20945/2359-3997000000091

**Published:** 2018-10-01

**Authors:** Sarah Crestian Cunha, Juliana Gabriel Ribeiro de Andrade, Camila Matsunaga de Angelis, Athanase Billis, Joaquim Murray Bustorff-Silva, Andréa Trevas Maciel-Guerra, Márcio Lopes Miranda, Gil Guerra-Júnior

**Affiliations:** 1 Universidade Estadual de Campinas Universidade Estadual de Campinas Faculdade de Ciências Médicas Departamento de Cirurgia Campinas SP Brasil Divisão de Cirurgia Pediátrica, Departamento de Cirurgia, Faculdade de Ciências Médicas, Universidade Estadual de Campinas (FCM-Unicamp), Campinas, SP, Brasil; 2 Universidade Estadual de Campinas Universidade Estadual de Campinas Faculdade de Ciências Médicas Grupo Interdisciplinar de Estudos da Determinação e Diferenciação do Sexo Campinas SP Brasil Grupo Interdisciplinar de Estudos da Determinação e Diferenciação do Sexo (GIEDDS), Faculdade de Ciências Médicas, Universidade Estadual de Campinas (FCM-Unicamp), Campinas, SP, Brasil; 3 Universidade Estadual de Campinas Universidade Estadual de Campinas Faculdade de Ciências Médicas Departamento de Anatomia Patológica Campinas SP Brasil Departamento de Anatomia Patológica, Faculdade de Ciências Médicas, Universidade Estadual de Campinas (FCM-Unicamp), Campinas, SP, Brasil; 4 Universidade Estadual de Campinas Universidade Estadual de Campinas Faculdade de Ciências Médicas Departamento de Genética Médica Campinas SP Brasil Departamento de Genética Médica, Faculdade de Ciências Médicas, Universidade Estadual de Campinas (FCM-Unicamp), Campinas, SP, Brasil; 5 Universidade Estadual de Campinas Universidade Estadual de Campinas Faculdade de Ciências Médicas Unidade de Endocrinologia Pediátrica Campinas SP Brasil Departamento de Pediatria, Unidade de Endocrinologia Pediátrica, Faculdade de Ciências Médicas, Universidade Estadual de Campinas (FCM-Unicamp), Campinas, SP, Brasil

## Abstract

A gonadal tumor was diagnosed in the first months of life in a patient with genital ambiguity, a 45,X/46,XY karyotype, and mixed gonadal dysgenesis. Gonadal biopsies at the age of 3 months revealed dysgenetic testes and a gonadoblastoma on the right testis. Even though gonadal tumors are rare in childhood, this case indicates that prophylactic removal of dysgenetic gonads should be performed as early as possible, especially when the female sex is assigned to a patient with a Y-chromosome sequence.

## INTRODUCTION

**D**isorders of sex development (DSD) are a group of congenital conditions in which the chromosomal, gonadal, or anatomical sex is atypical. These conditions include disorders of gonadal development, comprising various types of gonadal dysgenesis (GD) (1).

A GD may be either complete (CGD), with bilateral streak and female internal and external genitalia in XX or XY individuals; partial (PGD), characterized by genital ambiguity and two dysgenetic testes or a dysgenetic testis and a streak gonad in 46,XY subjects; or mixed (MGD), when a 45,X/46,XY karyotype or its variants are found in a patient whose gonadal and genital phenotype is similar to that of PGD (2).

In MGD, the presence of a 45,X cell line is associated with features of Turner syndrome, including short stature, cardiac and renal malformations, and thyroid disease, among others; thus, MGD and PGD have different prognoses (3,4).

The presence of Y-chromosome sequences in patients with GD increases their risk of developing gonadal tumors (5,6). In patients with MGD, this risk is estimated to be 3-4% and 10-20% at the ages of 10 and 15 years, respectively. The overall risk is 20-25% and may rise up to 46% at the age of 40 years (7).

In patients with MGD reared as girls, bilateral gonadectomy should be performed in childhood or upon diagnosis because of the risk of malignancy and insufficient gonadal function. For those reared as boys, removal of streak gonads and strikingly dysgenetic testes should also be done early; testes with lesser degrees of dysgenesis may be preserved in the scrotum and closely followed up for early detection of tumor development (8-11).

The aim of this report is to describe a patient with MGD who developed a gonadoblastoma within the first months of life. This case can add knowledge to support the decision about early prophylactic gonadectomy in this group of patients.

## CASE REPORT

A 43-day-old infant without sex assignment was referred to us due to genital ambiguity. This infant was born at term by cesarean section and without neonatal complications, with a birth weight of 2,580 g and a length of 45 cm. The infant – the only child of healthy, unrelated parents – also had a healthy, 17-month-old maternal half-brother.

The physical examination revealed a healthy infant with normal growth and without dysmorphic features, except for those of the external genitalia. A 3.5-cm phallus with chordee and penoscrotal hypospadias was present, and both gonads were palpable in the inguinal region. The features of the external genitalia were compatible with Prader grade III/IV (12), and the external masculinization score (EMS) (13) was 6.0.

The infant's karyotype was 45,X[7]/46,XY[43] and, on pelvic ultrasound, uterus and vagina were visualized. Basal levels of gonadotropins (FSH: 4.87 IU/L; LH: 5.45 IU/L), total testosterone (2.51 ng/mL), androstenedione (8.76 mg/dL), estradiol (22 pg/mL), and 17-hydroxyprogesterone (216.6 ng/dL) were normal for the reference values at minipuberty (when the hormonal values are similar to those of normal puberty). Abdominal ultrasound showed no urinary tract malformations, and echocardiography was also normal.

**Figure 1:**
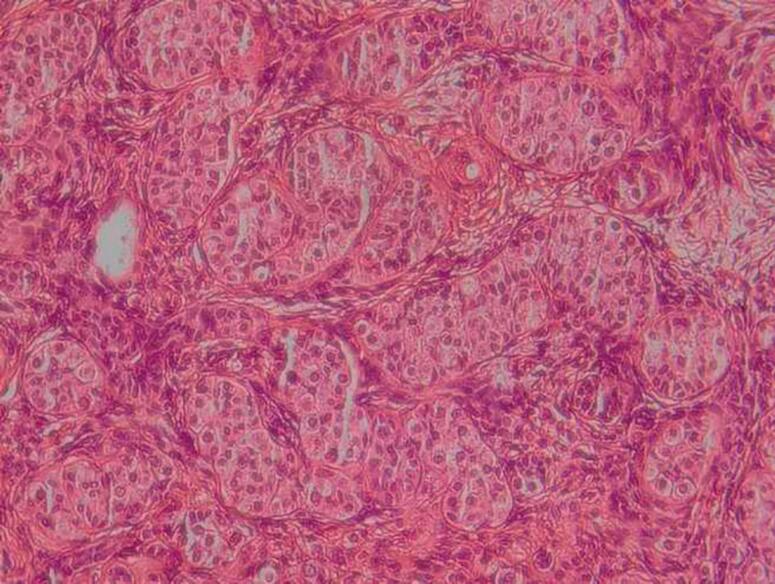
Biopsy of the right gonad showing germ cells intermingled with seminiferous tubules with decreased diameter.

Bilateral inguinotomy and biopsies of both gonads were performed at the age of 3 months to evaluate the degree of GD or the presence of ovarian tissue. At the same time, laparoscopy – which is the gold standard for the evaluation of internal genitalia (14) – confirmed the presence of uterus and fallopian tubes. The histology revealed bilateral dysgenetic testes with signs of neoplastic transformation on the right gonad (Figure 1).

**Figure 2:**
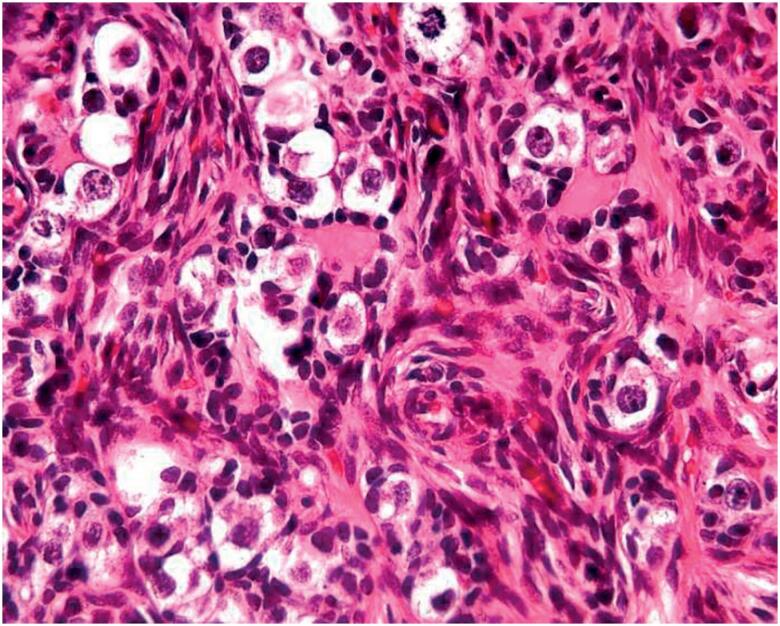
Large clear germ cells with prominent nuclei interspersed by other small cells with hyperchromatic nuclei and scant cytoplasm (immature Sertoli cells).

**Figure 3:**
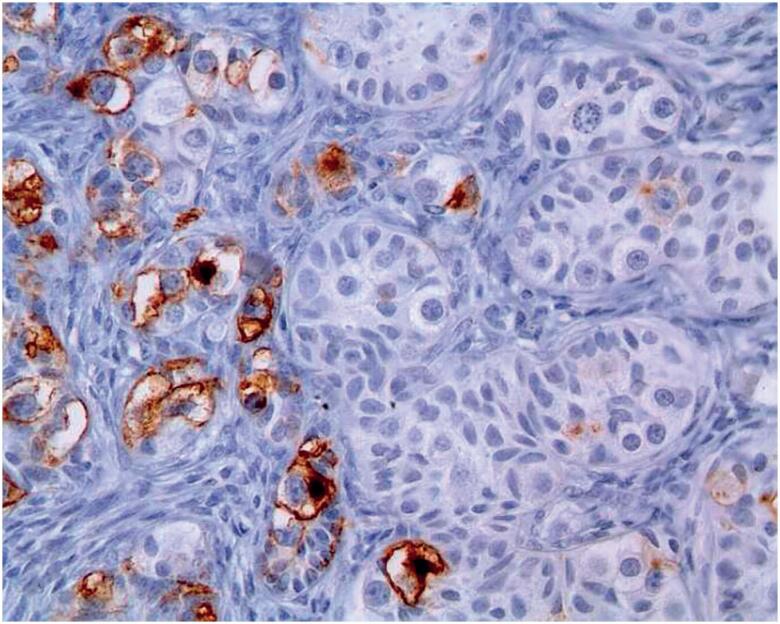
Germ cells positive for OCT3/4.

Based on the diagnosis of MGD, the anatomy of the internal and external genitalia, and the wish of the parents, the female gender was assigned to the infant, and bilateral gonadectomy was performed. The histological evaluation showed signs of bilateral testicular dysgenesis (areas of undifferentiated gonadal tissue, immature tubules surrounded by fibrosis together with well-differentiated testicular tissue, intracapsular tubules). The right gonad had two neoplastic lesions: a premalignant one (germ cell cluster, clonal proliferation), within a region of undifferentiated gonadal tissue, and a gonadoblastoma (Figures 2 and 3).

When the infant was 12 months old, a feminizing genitoplasty was successfully performed. She is currently 4 years old, well-adapted to the female social gender, and has a normal growth and neurological development.

## DISCUSSION

The finding of a 45,X/46,XY karyotype in patients with genital ambiguity and a female internal genitalia opens up the possibility for two differential diagnoses: MGD or ovotesticular DSD (1). The absence of ovarian tissue and the presence of bilateral dysgenetic testes in the patient presented here confirmed the diagnosis of MGD (1).

Even though in the past patients with bilateral testicular dysgenesis have been histologically classified as having PGD (2,5), the classification is now based on the infant's karyotype, and patients with a 45,X cell line are now diagnosed as having MGD (1,3). This classification is undoubtedly more useful, because the presence of a 45,X lineage prompts the evaluation of features of Turner syndrome: short stature, associated congenital anomalies (heart, kidney, and urinary system), as well as other associated diseases (autoimmune thyroid disease and hearing loss, among others) (3). The use of histological features for the classification may be misleading, due to the frequent limitation in evaluating the entire gonad. Therefore, the diagnosis of MGD in this case is in line with the DSD Consensus (1).

The most relevant aspect of this report is the presence of a gonadoblastoma in the right gonad, which was initially suspected at the age of 3 months and confirmed after the removal of the gonads. As of now, only a few cases of gonadal tumors have been described in very young patients with testicular dysgenesis. These include a carcinoma *in situ* in a 2-year-old boy with ambiguous genitalia, a 45,X/46,XY karyotype, and a dysgenetic right inguinal testis (4); bilateral gonadoblastoma in a 3-year-old girl with female genitalia, 46,XY karyotype, and bilateral dysgenetic gonads (6); and a left abdominal mass compatible with seminoma in a 15-day-old girl with ambiguous genitalia, 46,XY karyotype, and a right testis (15). In addition, bilateral gonadoblastoma has also been reported in a 7-month-old 46,XY girl with mutations in the *WT1* gene, dysgenetic right testis, and chronic renal failure in the first year of life; the original histology of the left gonad in the patient was undetermined (16).

Despite the usual benign behavior of gonadoblastomas, malignant transformation to seminomatous and nonseminomatous tumors can occur over the years. Other types of germ cell tumors associated with gonadoblastomas may be metastatic: in half of the cases, the tumor cells invade the stroma and turn into a dysgerminoma or seminoma (8). About 26% of all gonadoblastomas arise from dysgenetic gonads, and 20% of them emerge from cryptorchid dysgenetic testes, but in 54% of cases, the original structure of the gonad is hidden by the tumor (9).

Recently, Pleskacova and cols. (10) have defined risk groups for the development of gonadal tumors in GD according to the gonadal location, histopathology, and immunohistochemistry, as shown in Table 1.

**Table 1:** Groups with dysgenetic gonads at risk for the development of gonadal tumors (10)

	Low	Medium	High
Degree of virilization	Male genitalia	Slight undervirilization	Ambiguous genitalia
Gonadal location	Scrotal	Inguinal	Abdominal
Histopathology	Dysgenetic gonad without germ cell or ovary or dysgenetic testis with negative OCT3/4	Dysgenetic testis positive OCT3/4	Undifferentiated gonadal tissue or dysgenetic testicle positive OCT3/4
Immunohistochemistry	Negative OCT3/4	Positive OCT3/4 dispersed in the gonad, negative or weakly positive TSPY, negative stem cell factor, age less than 1 year	Positive OCT3/4 located in the basal lamina, focal location, strongly positive TSPY, positive stem cell factor, age more than 1 year

Concerning the present case, although the gonad was within the inguinal canal and the child was less than 1 year old, there were other features compatible with high-risk criteria for malignancy (ambiguous genitalia, dysgenetic testis, and positive and focal OCT3/4).

In conclusion, this rare case with an early neoplastic transformation confirms the need to carry out prophylactic gonadectomy in MGD patients as early as possible, particularly when the female gender is assigned to the infant. 
